# Supplementary material for: SumVg: Total Heritability Explained by All Variants in Genome-Wide Association Studies Based on Summary Statistics with Standard Error Estimates
Source: Int J Mol Sci. 2024 Jan 22;25(2):1347. doi: 10.3390/ijms25021347 (PMC10816209; doi:10.3390/ijms25021347)
Supplement: Supplementary file 1 [file ijms-25-01347-s001.zip › Figure S1 Bar plot of number of pruned SNPs, the sum of Vg, and SE approaches for 12 immune traits.pdf]

Figure S1 Bar plot of number of pruned SNPs, the sum of variance explained, and SE estimated by different approaches for 12 immune traits (under different r^2 pruning thresholds)

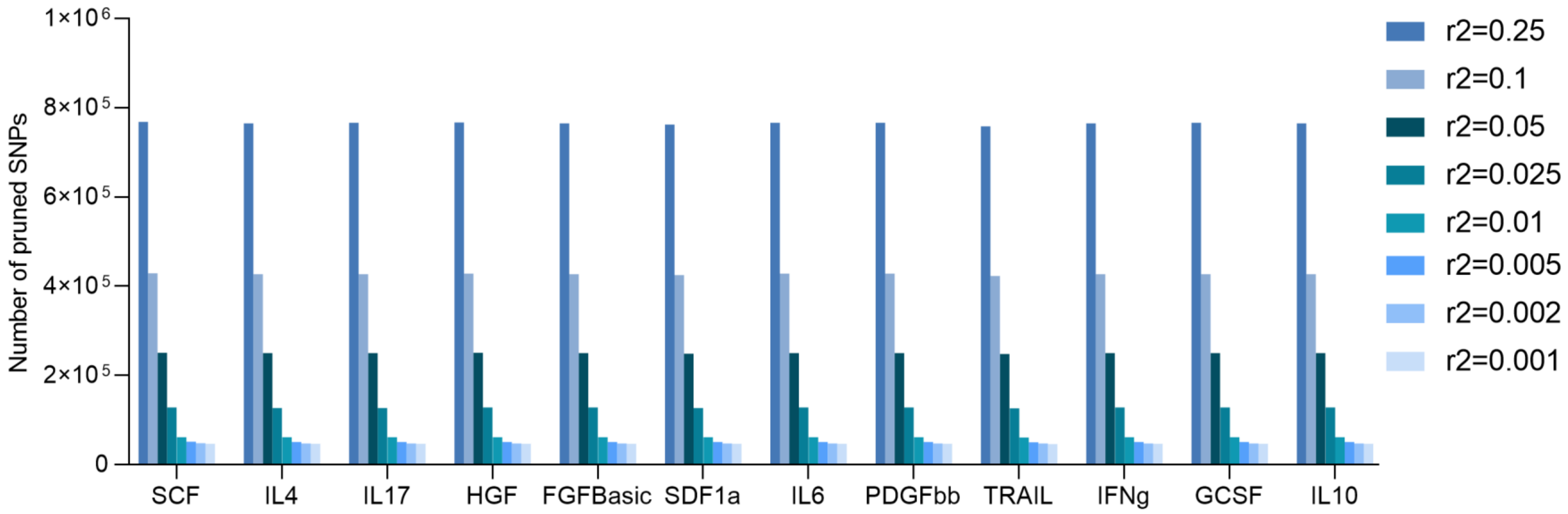

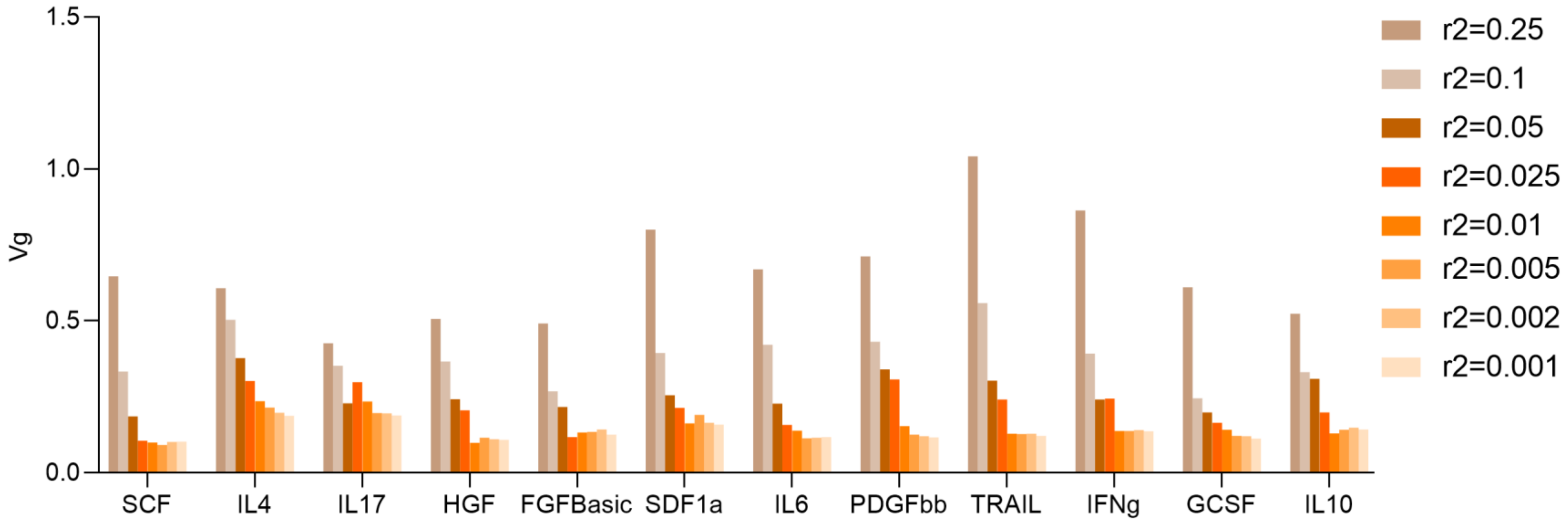

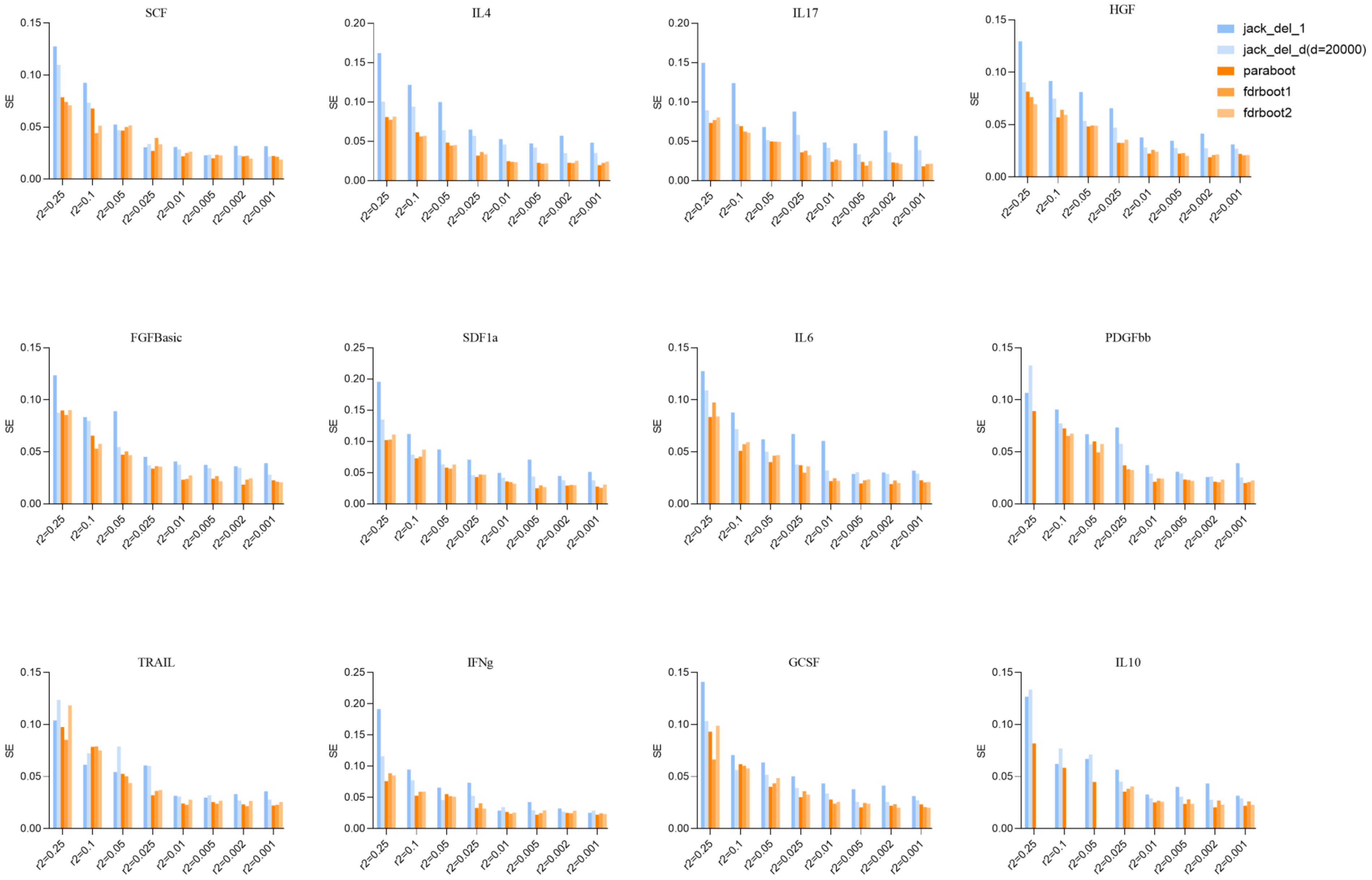

r2 is the pruning threshold, and Vg is the sum of variance explained that was estimated by SumVg. jack_del_1, jack_del_d, paraboot, fdrboot1 and fdrboot2 are different SE estimation approaches as described above.
